# Pan‐cancer population pharmacokinetics and exposure‐safety and ‐efficacy analyses of atezolizumab in patients with high tumor mutational burden

**DOI:** 10.1002/prp2.685

**Published:** 2020-11-25

**Authors:** Colby S. Shemesh, Phyllis Chan, Fatema A. Legrand, David S. Shames, Meghna Das Thakur, Jane Shi, Lorna Bailey, Shweta Vadhavkar, Xian He, Wei Zhang, René Bruno

**Affiliations:** ^1^ Clinical Pharmacology Genentech Inc. South San Francisco CA USA; ^2^ Clinical Science Genentech Inc. South San Francisco CA USA; ^3^ Biomarkers Genentech Inc. South San Francisco CA USA; ^4^ Safety Science F. Hoffmann‐La Roche Ltd. Shanghai China; ^5^ Safety Science Roche Products Ltd. Welwyn Garden City United Kingdom; ^6^ Biometrics Genentech Inc. South San Francisco CA USA; ^7^ Clinical Pharmacology Genentech‐Roche Marseille France

**Keywords:** atezolizumab, biomarkers, clinical pharmacology, mutation, pharmacokinetics, tumor

## Abstract

We retrospectively investigated the pharmacokinetics and exposure‐efficacy/safety relationships of single‐agent atezolizumab based on tissue tumor mutational burden (tTMB) status (high vs low [≥16 vs <16 mutations/megabase]) in a pan‐tumor population from seven clinical trials. Data sources included the OAK, POPLAR, BIRCH, FIR, IMvigor210, IMvigor211, and PCD4989g studies; 986 of 2894 treated patients (34%) had TMB data. Exposure metrics were obtained using a prior two‐compartment intravenous‐infusion population‐pharmacokinetics model, merged with prognostic, biomarker, efficacy, and safety variables. Baseline demographic/clinical characteristics and prognostic factors were well balanced between patients with high (n = 175) and low (n = 811) tTMB. Exposure was similar in the high‐ and low‐tTMB subgroups, with no difference seen in the evaluable vs total treated populations. The objective response rate (ORR) was 29.7% vs 13.4%, complete response rate was 6.9% vs 3.2%, and median duration of response (95% CI) was 29.0 (18.6‐NE) months vs 15.9 (12.5‐20.5) months for patients with high‐tTMB vs low‐tTMB tumors, respectively. A flat exposure‐efficacy relationship was seen for ORR in patients with high‐tTMB based on the cycle 1 minimum atezolizumab concentration and area under the serum concentration time curve (AUC). A nonsignificant exposure‐safety profile was seen for grade 3/4 adverse events and adverse events of special interest based on the AUC of atezolizumab in the high‐tTMB population. tTMB is an additional predictive biological factor affecting response to atezolizumab, and quantitative investigations of atezolizumab exposure and relationships of exposure with safety and efficacy support the use of a 1200‐mg, every 3‐week regimen in a tumor‐agnostic high‐tTMB population.

## Graphical abstract

In this work, we show that tissue tumor mutational burden (tTMB) is a biomarker of efficacy with the immune checkpoint inhibitor atezolizumab. The analyses herein support a role for atezolizumab monotherapy in a tumor‐agnostic high‐tTMB population.

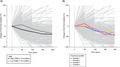

**Abbreviations**
CWRES: conditional weighted residuals
IC: tumor‐infiltrating immune cells
ICI: immune checkpoint inhibitor
mUC: metastatic urothelial carcinoma
NSCLC: non‐small cell lung cancer
pcVPC: prediction‐corrected visual predictive check
TC: tumor cells
TMB: tumor mutational burden
TNBC: triple‐negative breast cancer
tTMB: tissue tumor mutational burden

## 1 INTRODUCTION

Numerous clinical investigations of therapies that mobilize the immune system against cancer are underway, including those involving immune checkpoint inhibitors (ICIs), cell‐ or gene‐based therapies, oncolytic viruses, vaccines, targeted therapies, and other novel modalities.^1^, ^2^ Programmed death‐ligand 1 (PD‐L1)– and programmed death‐1 (PD‐1)–targeting ICIs prevent inhibitory signals to T cells, resulting in tumor rejection. These agents have expanded the therapeutic approaches in immuno‐oncology, inspiring >2250 trials in ICIs since 2018.^3^, ^4^ Within this expansion, biomarker‐based selection approaches, such as microsatellite instability/mismatch repair deficiency and PD‐L1 expression, are being developed to help guide the selection of ICI‐based therapies.^5^, ^6^ The US Food and Drug Administration (FDA) approval of ICI pembrolizumab for microsatellite instability‐high/mismatch repair–deficient cancers illustrated a paradigm shift^7^ ^,^ paving the way for other biomarker‐based tumor agnostic indications, including the mid‐2020 accelerated FDA approval of pembrolizumab in previously treated TMB‐high solid tumors.^8^

TMB reflects the number of somatic mutations existing per coding area of a tumor genome. The number of mutations can vary across tumor type, and many mutagenic processes can drive high TMB, including but not limited to DNA replication infidelity, mismatch repair deficiency, environmental mutagens such as tobacco smoke and ultraviolet light, contaminated food pathogens, and aging.^9^ Nonsynonymous mutations increase the number of tumor‐specific neoantigens recognized by the immune system, thus, TMB is a proxy estimate of the neoantigen load of a tumor.^10^ This process increases the number of tumor‐infiltrating immune cells (IC) in the tumor microenvironment and bolsters cytotoxic T‐cell responses. TMB was found to correlate with response to ICIs in a cross‐study analysis of 27 tumor types^11^ and in a prospective multicohort evaluation,^12^ and correlations with overall survival have further reinforced the predictive value of TMB in many cancers.^11^, ^13^, ^14^, ^15^ Given the association of TMB with response to ICIs, the substantial number of ongoing clinical trials surveying TMB as a potential biomarker is unsurprising.^13^

Atezolizumab is an anti–PD‐L1 monoclonal antibody that selectively targets PD‐L1 to inhibit interaction with its receptors PD‐1 and B7.1 to enhance T‐cell responses.^16^ Atezolizumab is approved by a number of global health authorities, as monotherapy or in combination with other agents, across several tumor types, including metastatic urothelial carcinoma, metastatic nonsquamous non‐small cell lung cancer (NSCLC), metastatic triple‐negative breast cancer (TNBC), and extensive‐stage small cell lung cancer,^17^, ^18^, ^19^, ^20^, ^21^, ^22^, ^23^ among others. Atezolizumab is also under investigation for patients with previously treated solid tumors with high TMB, including a prospective clinical trial (NCT02091141).

Despite increases in ICI approvals and quantitative clinical pharmacology characterizations of atezolizumab and other ICI agents, no histologically independent empirical analyses of the clinical pharmacology of ICI‐based therapies as a function of TMB have been performed.^25^, ^26^ Therefore, we evaluated tissue TMB (tTMB) as a predictive biomarker and describe both the clinical outcomes and the in‐depth clinical pharmacology of atezolizumab monotherapy for patients with high‐tTMB tumors of several cancer types from seven phase I, II, and III studies.

## 2 MATERIALS AND METHODS

### 2.1 Studies contributing to the tTMB analysis

Seven studies evaluating the efficacy and safety of atezolizumab monotherapy were included in this analysis, as described in Table S1: (a) OAK (ClinicalTrials.gov ID NCT02008227)—a phase III, open‐label, randomized study of atezolizumab vs docetaxel in platinum‐treated NSCLC, (b) POPLAR (NCT01903993)—a phase II, open‐label, randomized study of atezolizumab vs docetaxel in platinum‐treated NSCLC; (c) BIRCH (NCT02031458) and (d) FIR (NCT01846416)—both phase II, open‐label, single‐arm studies of atezolizumab in PD‐L1–selected locally advanced or metastatic NSCLC; (e) IMvigor211 (NCT02302807)—a phase III, open‐label, randomized study of atezolizumab vs chemotherapy in platinum‐treated locally advanced or metastatic urothelial carcinoma; (f) IMvigor210—a phase II, open‐label, single‐arm study of atezolizumab in previously untreated (NCT02951767) or platinum‐treated (NCT02108652) metastatic urothelial carcinoma; and (g) PCD4989g—a first‐in‐human, phase I, open‐label, dose‐escalation study (NCT01375842) of atezolizumab as a single agent in locally advanced or metastatic solid tumors or hematologic malignancies. Only atezolizumab monotherapy studies were included to limit any potential bias from combination agents. PD‐L1 status was evaluated using the VENTANA SP142 immunohistochemistry assay (Ventana Medical Systems, Tucson, Arizona).

The phase II/III studies each used a 1200‐mg dose of atezolizumab every 3 weeks (q3w). The phase I study PCD4989g also included some patients treated with 10‐, 15‐, and 20‐mg/kg doses of atezolizumab q3w, and these patients were also included in this analysis. Atezolizumab was administered by intravenous infusion on day 1 of each 21‐day cycle (infusion duration, 60 minutes in cycle 1 and 30 minutes in subsequent cycles if no infusion‐related adverse events [AEs] were observed).

Each study was conducted in accordance with the Declaration of Helsinki and Good Clinical Practice Guidelines, following ethics board approval at each institution. Informed consent was obtained from each patient.

### 2.2 tTMB assessment

tTMB was evaluated by the FoundationOne hybrid‐capture next‐generation‐sequencing assay (F1). Details on the F1 platform and TMB‐estimation algorithms were previously reported.^27^, ^28^, ^29^ Briefly, the assay detects substitutions, insertion, deletion alterations, and copy number alterations in 324 genes using DNA from formalin‐fixed paraffin‐embedded solid tumor specimens. The number of somatic mutations is quantified as mutations per megabase (mut/Mb) by removing polymorphisms and predicted drivers from all variants to provide somatic mutation count per Mb. The distribution of tTMB was observed to be a continuous variable, with a median tTMB of 7.9 mutations/Mb. To determine an appropriate cutoff from the retrospective analysis, a tTMB‐high cutoff was established based on balancing between a high response rate and a reasonable prevalence across a heterogenous set of tumor types (see Figure S1). A cutoff of ≥16 mutations/Mb was selected. Pooled response rates were evaluated at tTMB cutoffs from 4 to 24 mutations/Mb in the tTMB‐evaluable population (n = 986) across the evaluated studies. The results revealed an enrichment of response rates with increasing TMB in the pooled data set. Patients evaluable for tTMB were retrospectively grouped based on a ≥16‐mutations/Mb cutoff for high‐tTMB—established with the use of a prior receiver operating characteristic assessment.^24^ The relationship with tTMB and objective response rate (ORR per investigator‐assessed Response Evaluation Criteria in Solid Tumors [RECIST] version 1.1,^30^) duration of response (DOR), and incidences of grade 3/4 AEs and any‐grade AEs of special interest (AESIs) were evaluated. Although blood TMB was not collected across all studies in this analysis, a positive correlation between tTMB and matched bTMB scores was previously demonstrated in the OAK and POPLAR studies, suggesting that tTMB data herein would be concordant.^31^

### 2.3 Pharmacokinetics (PK) sampling and analytical methods

In POPLAR, BIRCH, FIR, OAK, IMvigor210, and IMvigor211, PK sampling of atezolizumab occurred as follows: following infusion on day 1 of cycle 1; prior to infusion on day 1 of cycles 1, 2, 3, 4, 8, and 16; every eight cycles thereafter; at discontinuation; and 120 days after the last dose. The phase I study followed the same scheme as above, but additional samples were collected from the majority of patients at cycle 1 (24 hours, 72 hours, day 8, and day 15) and pre‐dose (prior to infusion) at cycles 5, 7, 10, 12, and 14. Blood samples from patients were centrifuged at 1500‐2000*g* for 15 minutes at 4°C. The serum samples were then stored at −60°C or less. Atezolizumab concentrations were quantified by enzyme‐linked immunosorbent assay (ELISA), with a 60‐ng/mL lower limit of quantification in human serum. The method for measuring atezolizumab in human serum was validated and included an inter‐run and intra‐run precision (%coefficient of variation [%CV]) of ≤4.59% and ≤4.12%, and inter‐run and intra‐run accuracy (%relative error) of −7.13% to 4.17% and −7.17% to 3.96%, respectively. The assay specifically detected atezolizumab in disease stage samples. No interference was observed from hemolysis, lipemia, and co‐medications.

### 2.4 Population‐PK model and derivation of exposure metrics

A previously developed two‐compartment population‐PK (popPK) model of atezolizumab based on phase I (PCD4989g) data^32^ was used in the PK analyses. According to the Phase I popPK model, the typical clearance (CL, in L/day) of atezolizumab for patient *i* was:

$${\text{CL}}_{i}\;=\;\left(0.200\;\times \;{\left(\frac{{\text{ALBU}}_{i}}{40}\right)}^{‐1.12}\;\times \;{\left(\frac{{\text{BWT}}_{i}}{77}\right)}^{0.808}\;\times \;{\left(\frac{\text{Tumor}\;{\text{burden}}_{i}}{63}\right)}^{0.125}\right)\;\times \;\left(1\text{.159}\;\;\text{if}\;\text{ADA}\;\text{is}\;\text{positive}\right),$$

where BWT = body weight (kg); ALBU = albumin (g/L); Tumor burden (mm); ADA = post‐baseline status of anti‐drug antibodies: post‐baseline negative anti‐drug antibody (ADA) when post‐dose samples after baseline or ADA signal not enhanced after baseline (treatment unaffected); post‐baseline positive ADA when treatment induced or treatment enhanced; and missing when all post‐dose samples missing. The typical volumes of distribution of the central compartment (V1) (L) and the peripheral compartment (V2) (L) of atezolizumab for patient *i* were:

$$V1_{i}=\left(3.28\times {\left(\frac{{\text{BWT}}_{i}}{77}\right)}^{0.559}\times {\left(\frac{{\text{ALBU}}_{i}}{40}\right)}^{‐0.350}\right)\times \left(0.871\;\text{if}\;\text{female}\right)$$

$$V2_{i}\;=\;3.63\;\times \;\left(0.728\;\;\text{if}\;\;\text{female}\right).$$

A combined model with proportional and additive components describes the residual error.

The popPK model was developed based on the Phase I study, which used a more intensive PK sampling schedule than the Phase II and III studies, from which mostly trough PK samples were collected. Therefore, the popPK model was not re‐developed and the parameters were not re‐estimated based on the pooled data. Monotherapy data pooled across studies were used to validate and externally evaluate the performance of the model. The popPK analysis was performed with NONMEM v7.4 (ICON Development Solutions) in conjunction with Perl‐Speak‐NONMEM (PsN) (v3.7.6; Uppsala University).

When covariate values in the pooled data set were missing in <15% of the total number of patients, the values were imputed to median values for continuous covariates or to the most frequent category for categorical covariates. Performance of the phase I popPK model on the current data set was evaluated at the population level, without fitting the data, by several goodness‐of‐fit plots: observed dependent variable (atezolizumab concentration) and conditional weighted residuals (CWRES) vs population predictions, CWRES vs time, quantile‐quantile plot of CWRES, random‐effect distributions, and correlations of random effects between parameters. The predictive performance of the popPK model was also evaluated with a simulation‐based prediction‐corrected visual predictive check (pcVPC). Based on popPK parameter estimates of the Phase I popPK model, profiles for atezolizumab concentrations vs time were simulated in 1000 replicates of studies with the same design as the studies included in this evaluation.^33^, ^34^ Bayesian estimation of individual PK parameters (MAXEVAL = 0 in NONMEM) was used to compute atezolizumab exposure variables based on the nominal dose regimen, including area under the curve (AUC), maximum concentration (*C*_max_), and minimum concentration (*C*_min_) in cycle 1 and beyond, including steady state. Derivation of cycle 1 *C*_max_ was based on day 1 of cycle 1 post‐infusion samples, while cycle 1 *C*_min_ was based on day 1 of cycle 2 pre‐dose samples.

### 2.5 Exposure‐efficacy analysis

Patients with measurements of tTMB, sum of longest diameter (SLD; of target lesions, at baseline and every other cycle), and atezolizumab cycle 1 *C*_min_ were included. Tumor scans were obtained on days 0, 63, 126, 189, and 252. Mean percentage change in tumor SLD from baseline was evaluated in the pooled population in tTMB‐low and tTMB‐high patient subgroups and by the quartile of cycle 1 atezolizumab *C*_min_ exposure. SLD change overall and by exposure quartile comparisons may be biased and underestimated on the account of discontinuation differences due to disease progression. For both the exposure‐efficacy and ‐safety analyses, first treatment cycle exposure metrics were used rather than steady‐state metrics to avoid confounding factors on exposure, such as response‐dependent time‐varying clearance.^26^ *P* values were calculated for exploratory purposes only. The relationship between the probability of achieving an objective response and atezolizumab exposure was investigated by logistic regression as well as graphically, by dividing exposure metrics into quartiles and plotting the observed (95% CI) ORR against the median exposure metric of each quartile; bootstrapped replicates (n = 100) were used to plot the 95% confidence band for the mean fit curve. SLD and RECIST 1.1 objective response were used as measures of efficacy for exposure‐response analyses because: (1) they directly reflect tumor growth patterns following treatment with atezolizumab, whereas later time‐to‐event outcomes, such as OS, may be confounded by intervening treatments and (2) not all studies in this analysis employed a comparator arm in their trial design, rendering selection of an appropriate clinical endpoint comparator for a heterogeneous pan‐tumor population challenging and potentially not feasible given the inherent assumptions required. Indeed, clinical development with pembrolizumab's microsatellite instability and TMB biomarkers in pan‐tumor populations were previously supported by using ORR and DOR data.^7^

### 2.6 Exposure‐safety analysis

Patients for whom both tTMB measurements and exposure data were available were included. Exposure‐safety was assessed for grade 3/4 AEs and any‐grade AESIs. Atezolizumab exposure levels were grouped based on quartiles of log‐transformed AUC and displayed as described for the probability of response.

### 2.7 Nomenclature of targets and ligands

Key protein targets and ligands in this article are hyperlinked to corresponding entries in http://www.guidetopharmacology.org, the common portal for data from the IUPHAR/BPS Guide to PHARMACOLOGY,^35^ and are permanently archived in the Concise Guide to PHARMACOLOGY 2019/20.^36^

## 3 RESULTS

### 3.1 Patient demographics and clinical characteristics

**FIGURE 1:**
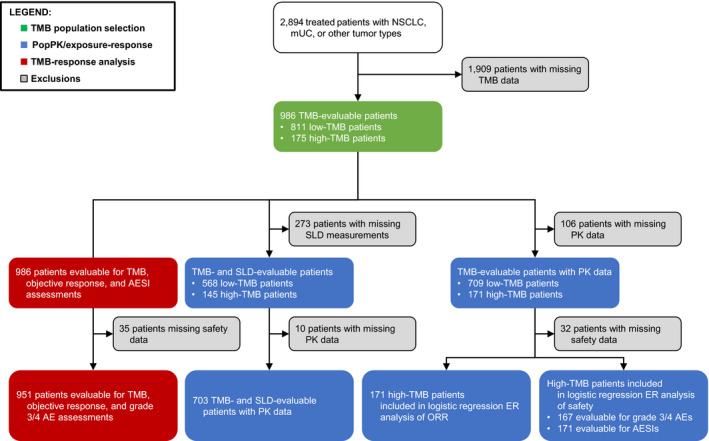
Flowchart of the analysis populations. AESI, adverse event of special interest; ER, exposure‐response; mUC, metastatic urothelial carcinoma; NSCLC, non‐small cell lung cancer; popPK, population pharmacokinetics; SLD, sum of longest diameters; TMB, tumor mutational burden

The pooled data set of tTMB‐evaluable patients comprised 986 patients (of 2894 treated patients; 34.1%), of whom 811 had low tTMB (<16 mut/Mb) and 175 had high tTMB (≥16 mut/Mb). Patient characteristics by tTMB status are summarized in Table 1. The tTMB‐low population had ≥16 tumor types, while the tTMB‐high population had ≥8 tumor types (Table S2). Median baseline SLD was similar in both groups (60 and 58 mm, respectively). In both groups, the median number of metastatic sites was two, and median Eastern Cooperative Oncology Group performance status (ECOG PS) was 1. The percentage of tumors with PD‐L1 TC2/3 and/or IC2/3 status (PD‐L1 expression on ≥5% of tumor cells [TC] and/or IC, respectively) was slightly lower in tTMB‐low patients than in tTMB‐high patients (18.2% and 40.8% vs 25.7% and 47.4%, respectively). The median C‐reactive protein levels were approximately 20% lower in tTMB‐low patients than tTMB‐high patients, while the neutrophil‐to‐lymphocyte ratio and albumin and lactate dehydrogenase levels were balanced between both groups. The median tTMB was approximately three‐fold lower in patients with low tTMB vs high‐tTMB. The analysis populations are depicted in Figure 1.

**TABLE 1:** Patient baseline demographic and clinical characteristics.^a^

Covariate	Pooled tTMB <16 mut/Mb (n = 811)	Pooled tTMB ≥16 mut/Mb (n = 175)	*P*‐value
Median age (range), years	65 (20‐88)	65 (37‐89)	.1211
Median albumin (range), g/L	40 (0.035‐270)	39 (0.039‐49)	.3577
Anti‐drug antibody positive, n (%)	216 (32.9)	48 (29.3)	.3791
Missing records, n (%)	154 (19.0)	11 (6.3)	—
Median baseline SLD (range), mm	60 (10‐310)	58 (11.1‐309)	.5840
Median body weight (range), kg	74 (37‐149)	75 (35.4‐149)	.5018
White, n (%)	624 (76.9)	145 (82.9)	.7337
Median C‐reactive protein (range), mg/L	12.6 (0.26‐318)	15.6 (0.41‐288)	.0373*
Missing records, n (%)	223 (27.5)	22 (12.6)	—
Median ECOG PS (range)	1 (0‐2)	1 (0‐2)	.6248
Female, n (%)	307 (37.9)	48 (27.4)	.0092**
Median lactate dehydrogenase (range), U/L	223 (0.83‐3137)	209 (77‐1407)	.0521
Median prior lines of therapy (range)	2 (1‐4)	2 (1‐3)	.8991
Missing records, n (%)	99 (12.2)	19 (10.9)	—
Median neutrophil to lymphocyte ratio (range)	3.81 (0.865‐59.7)	4.00 (0.965‐46.5)	.3695
Median no. of metastatic sites (range)	2 (0‐7)	2 (0‐7)	.6914
Median no. of tumor types present^b^	16	8	—
PD‐L1 TC2/3, n (%)^c^	147 (18.2)	45 (25.7)	.0239*
Missing records, n (%)	5 (0.6)	—	—
PD‐L1 IC2/3, n (%)^d^	331 (40.8)	83 (47.4)	.1078
Median tTMB (range), mut/Mb	7.02 (0‐15.8)	22.8 (16.7‐403)	<.0001****

Note

Significance of the difference between patient groups at baseline was evaluated using a Chi‐square test for categorical variables, and Mann‐Whitney test for continuous variable, *P* < .05, *P* < .01, and *P* < .0001 are denoted by *, **, and **** respectively.

ECOG PS, Eastern Cooperative Oncology Group performance score; IC, tumor‐infiltrating immune cell; mUC, metastatic urothelial carcinoma; NSCLC, non‐small cell lung cancer; PD‐L1, programmed death‐ligand 1; SLD, sum of longest diameter; TC, tumor cell; tTMB, tissue tumor mutational burden.

^a^ Distribution of patients by tTMB status included 50% NSCLC, 42.4% mUC, and 7.27% encompassing >13 tumor types.

^b^ See Table S2 for tumor types within each subgroup.

^c^ PD‐L1 expression on ≥5% of TC.

^d^ ≥5% of the tumor area occupied by PD‐L1–expressing IC per VENTANA SP142 immunohistochemistry assay.

### 3.2 PopPK model

A total of 2672 treated patients with exposure data were included in the popPK data set, excluding 223 unevaluable patients. Of the 880 patients evaluable for tTMB and exposure, 709 were categorized as tTMB low and 171 as tTMB high. A total of 14 596 samples were available for the tTMB‐evaluable patients. PK data were sparse (mean: 5.4 samples/patient), with fewer samples at *C*_max_ (cycles 1‐2) and more samples at *C*_min_.

Goodness‐of‐fit plots were adequate for population predictions (Figure S2A) of the pooled data without re‐estimation of the popPK model parameters. CWRES were homogeneously distributed around 0, suggesting no bias in the predictions of high and low concentrations of atezolizumab. The pcVPC was performed using *C*_max_ and *C*_min_ atezolizumab data stratified by tTMB level, as shown in Figure S3. The pcVPC plots suggested that the median, 5th, and 95th percentiles of observed *C*_max_ and *C*_min_ were within the prediction interval of the previously developed popPK model.

Having found that the popPK model described the data well, we estimated individual parameters by fitting the data in the pooled data set. The goodness‐of‐fit plots at individual levels, shown in Figure S2B, were also adequate for individual predictions. IWRES were homogeneously distributed around 0, suggesting no bias in the predictions of high and low concentrations of atezolizumab. Slight deviations are observed around certain extremes of the exposure or time range but are unlikely to be associated with model misspecification due to the sparse nature of these data.^37^ Inter‐individual variability relationships between random effects of central clearance, central volume of distribution, and peripheral volume of distribution in the popPK model using tTMB as a continuous variable indicated no significant effect on the popPK parameters (Figure S4).

### 3.3 Exposure metrics

**FIGURE 2:**
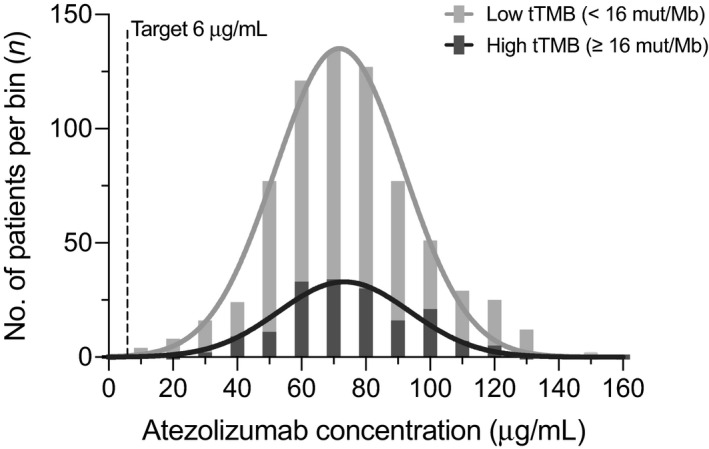
Atezolizumab exposure distribution by tTMB status. Post hoc analysis of exposures across 880 patients treated with atezolizumab 1200 mg are shown, including 709 patients with tTMB <16 mut/Mb and 171 patients with tTMB ≥16 mut/Mb. The dotted line indicates the therapeutic target exposure of 6 µg/mL. The height of the bar represents the number of patients within that concentration range, while the width represents binning of patients from 0 to 160 µg/mL by multiples of 10. A cumulative distribution trend (dark and light grey lines) is superimposed over the frequency distribution histogram for each subgroup. tTMB, tissue tumor mutational burden

The model‐derived geometric mean (%CV) cycle 1 *C*_min_ of atezolizumab was 62.6 µg/mL (212%) in the overall treated population (n* = *2672) and 66.0 µg/mL (134%) in the pooled tTMB‐evaluable population (n = 880). The corresponding values in the tTMB‐low (n = 709) and ‐high (n = 171) populations were 66.7 (120%) and 63.2 (200%) µg/mL, respectively. We summarized the model‐derived atezolizumab‐exposure metrics for 709 patients with low tTMB and 171 with high tTMB at cycle 1 and at steady state (Table 2). The large %CV associated with *C*_min_ are related to the low exposure levels observed in a small number of patients. The AUC, *C*_max_, *C*_min_, and clearance were each similar between both groups, with a maximal difference observed between groups of <1%. Figure 2 illustrates the distribution of cycle 1 *C*_min_ in patients with low tTMB vs high tTMB. The median patient exceeded the target trough exposure of 6 µg/mL^38^ by more than 10‐fold, regardless of tTMB status, with overlapping distributions between subgroups.

**TABLE 2:** Predicted summary statistics of atezolizumab‐exposure metrics

Metric	Observation	tTMB <16 mut/Mb (n* = *709)	tTMB ≥16 mut/Mb (n = 171)
*C*_max_, µg/mL	Cycle 1	376 (26.9)	367 (30.2)
	Steady state	546 (33.1)	532 (37.2)
*C*_min_, µg/mL	Cycle 1	66.7 (120)	63.2 (200)
	Steady state	154 (143)	144 (235)
AUC, µg∙day/mL	Cycle 1	2830 (47.4)	2722 (67.2)
	Steady state	5361 (61.5)	5142 (81.4)
CL, mL/day/kg	—	0.223 (62.1)	0.232 (82.1)

Note

Data in the table are geometric means (% coefficients of variation).

AUC, area under the curve; CL, clearance; *C*_max_, maximum concentration; *C*_min_, minimum concentration; tTMB, tissue tumor mutational burden.

### 3.4 TMB‐efficacy and exposure‐efficacy analysis

**FIGURE 3:**
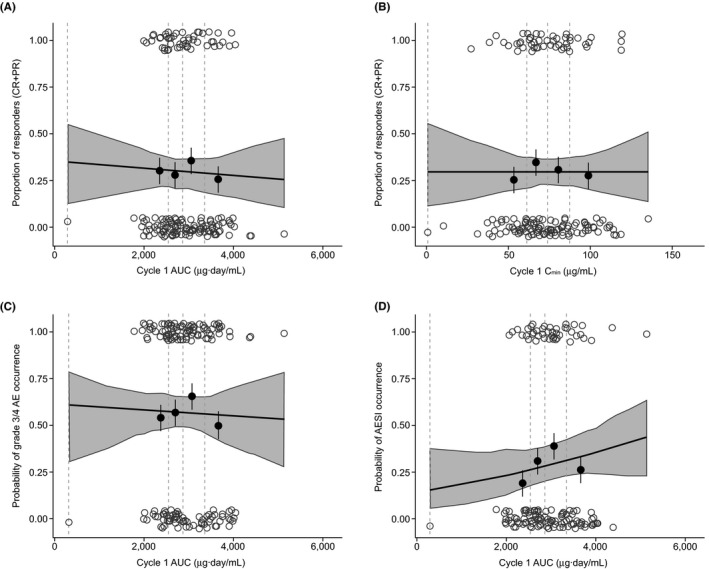
Proportion of tTMB‐high patients who were responders (CR + PR) to atezolizumab by (A) cycle 1 AUC and (B) cycle 1 *C*_min_ and the proportion of tTMB‐high patients with grade 3/4 AEs by AUC (C) and (D) any‐grade AESI by AUC. AUC and *C*_min_ values for each response event (yes, 1.00; no, 0) are represented by open grey circles. Solid black circles with standard error bars: proportion of response from binned observations by quartiles of the log‐transformed exposure (*y* value); median exposure value within the bin (*x* value). Black line: model‐fitted curve of the probability of response across atezolizumab exposure. Dashed lines: binning boundaries. Shaded area: 95% confidence band for the logistic regression curve. Observed data points are based on 171 and 167 patients for efficacy and safety, respectively. AESI, adverse event of special interest; AUC, area under the curve; *C*_min_, minimum concentration; CR, complete response; PR, partial response; tTMB, tissue tumor mutational burden

Among 986 tTMB‐evaluable patients, the ORR was 13.4% (109 of 811 patients) in the low‐tTMB group and 29.7% (52 of 175 patients) in the high‐tTMB group. The odds ratio (OR) for ORR by tTMB status, evaluated using a contingency table and Fisher exact test, revealed a statistically significant (*P* < .0001) OR of 2.72 (95% CI, 1.87‐4.01) for patients with tTMB‐high vs tTMB‐low status. This observation—that patients with high tTMB were more likely to achieve an objective response with atezolizumab than patients with low tTMB (ratio of over two odds)—confirms the positive association and predictive value between ORR and tTMB status that has been observed in other meta‐analyses following the use of ICIs.^39^ The corresponding complete response (CR) frequencies were 3.2% (26 of 811 patients) and 6.9% (12 of 175 patients) in the low‐tTMB and high‐tTMB subgroups, respectively. DOR by tTMB status is shown in Figure S5. Per investigator assessment, a 15.9‐month median DOR (95% CI, 12.5‐20.5) in low‐tTMB patients and 29.0‐month median DOR (95% CI, 18.6‐NE) in high‐tTMB patients was observed. A logistic regression evaluation showed that the probability of achieving an objective response in patients with high tTMB was not significantly correlated to atezolizumab AUC at cycle 1; the exploratory *P* value was 0.751 (Figure 3A). Similar results were found when cycle 1 *C*_min_ was used (Figure 3B, exploratory *P* = .998).

**FIGURE 4:**
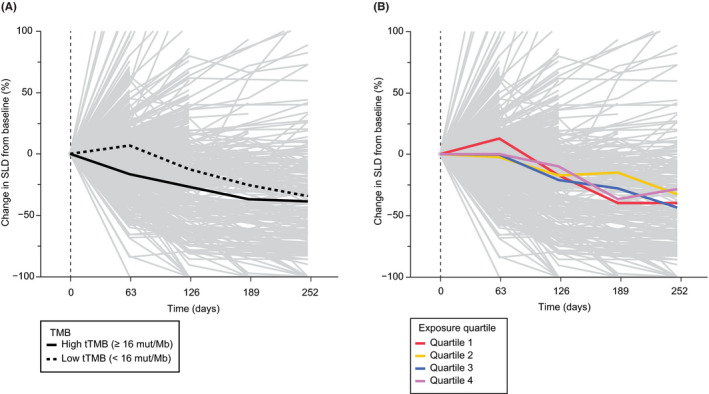
Change in SLD from baseline by TMB status and atezolizumab exposure. (A) Tumor size change across 713 tTMB efficacy‐evaluable patients with available tumor scan data (gray). Mean changes in tumor size from baseline are represented by the solid black line for the tTMB‐high subgroup (≥16 mut/Mb; n = 145) and by the dotted black line for the tTMB‐low subgroup (<16 mut/Mb; n* = *568). (B) tTMB efficacy and cycle 1 atezolizumab *C*_min_ PK‐evaluable patients (n = 703). Mean tumor size changes from baseline as a function of cycle 1 atezolizumab *C*_min_ exposure quartile are shown in red (n = 176), yellow (n = 176), blue (n = 176), and green (n = 175). *C*_min_, minimum concentration; PK, pharmacokinetics; SLD, sum of longest diameter; tTMB, tissue tumor mutational burden

The mean change in tumor size is shown by tTMB status in Figure 4A and by cycle 1 *C*_min_ exposure quartile in Figure 4B. More pronounced tumor shrinkage over time was seen in patients with high tTMB. At 18 weeks (126 days), the mean reduction in SLD from baseline was −26.8% in patients with high TMB compared with −12.5% in patients with low TMB. Tumor shrinkage in the low TMB population approached that in the high TMB population later during treatment, as progressors were inherently lost from the fitting line over time. Thus, while distinctions in efficacy by tTMB status were observed in the overall tTMB‐evaluable population, no trends with higher exposure quartiles of atezolizumab were seen.

### 3.5 Exposure‐safety analysis

The exposure‐safety analysis was performed in high‐tTMB patients with exposure data. Grade 3/4 AEs and all‐grade AESIs occurred at an incidence of 56.9% (events in 167 patients) and 40.4% (events in 171 patients), respectively. The incidence of grade 3/4 AEs and AESIs by atezolizumab cycle 1 AUC is shown in Figure 3C and D. Exposure metrics within the first treatment cycle were used rather than steady‐state metrics to isolate potentially confounding factors on exposure, such as time‐varying clearance.^26^ There was no consistent trend of increased AE incidence with increased exposure for both grade 3/4 AEs (exploratory *P* = .812) and AESIs (exploratory *P* = .280) with atezolizumab AUC. Similarly, no trend was detected with *C*_max_ or *C*_min_ (Figure S6). An analysis of exposure‐safety in the entire TMB biomarker–evaluable population further confirmed no meaningful exposure‐response trend. Lastly, safety in high‐tTMB patients was also comparable to that in all treated patients (grade 3/4 AEs, 55.7% [n = 1557/2794]; all‐grade AESIs, 30.9% [n = 894/2894]).

### 3.6 tTMB‐response analysis

Additional exploratory analyses were conducted to evaluate longitudinal relationships of efficacy and safety by tTMB in the pooled data set. Overall, a steep relationship was observed between an increasing tTMB and the proportion of responders (patients with CR + PR) following treatment with atezolizumab, whereas the tTMB‐response curve for grade 3/4 AE occurrence was flat and the tTMB‐response curve for all‐grade AESI occurrence was shallow (Figure S7).

### 3.7 Tumor types of responding patients with high tTMB

Response by tumor type for tTMB‐evaluable responding patients is shown in Table S3. In patients with high tTMB, objective responses occurred across four tumor types: urothelial carcinoma (UC; bladder cancer), endometrial, melanoma, or NSCLC; the nonresponsive tumor types comprised only 9 patients. Aside from the single patient with tTMB‐high endometrial cancer (n = 1) who had an objective response, response rates ranged from 28%‐42% for the other responding tumor types; albeit based on a small sample size (n = 12), patients with melanoma appeared to have numerically higher ORRs and CR rates (42% and 17%, respectively). For the 2 responsive tumor types represented in both tTMB subgroups, response rates were higher in tTMB‐high patients than tTMB‐low patients: 23 of 83 patients (28%) with high‐tTMB NSCLC and 23 of 70 patients (33%) with high‐tTMB UC had an objective response to atezolizumab, compared with 41 of 259 (16%) and 52 of 330 (16%) patients with low‐tTMB tumors, respectively. A CR was seen in 7 patients (10%) with high‐tTMB urothelial carcinoma cancer and 3 patients (4%) with high‐tTMB NSCLC compared with 17 (5%) and 5 (2%) respective patients with low tTMB.

## 4 DISCUSSION

As a result of widespread advances and expansion in immuno‐oncology, the development of novel immunotherapy biomarkers to improve patient selection represents an ongoing challenge, given that not all patients derive benefit from ICIs.^40^ Trials of ICIs evaluating TMB as a predictive biomarker have struggled to affect guideline recommendations on the use of TMB in clinical practice.^41^ Previous investigations revealed that tTMB—orthogonal to PD‐L1 expression—can serve as a complementary and/or alternative biomarker that may provide predictive value and address the unmet medical need in patient populations currently not being served by ICIs in a tumor‐agnostic manner.^24^

The characterization of prognostic factors, dose selection, and exposure‐response relationships is important in optimizing effective immunotherapies; yet, this area has been underinvestigated or has not been applied in a high‐TMB tumor‐agnostic indication.^42^, ^43^ Although tTMB is not expected to influence drug exposure, this is the first account of quantitative clinical pharmacology findings of an ICI evaluated by tTMB across multiple trials in patients with solid tumors. We observed that high tTMB is a potential positive predictive marker associated with increased clinical benefit following treatment with atezolizumab in diverse cancers—a finding similar to that in meta‐analyses of other PD‐L1/PD‐1 agents.^44^, ^45^ Baseline prognostic factors that may lead to bias should also be explored. Patient characteristics and prognostic factors important in assessing clinical impact were generally well balanced between patients by tTMB status. In our study a larger percentage of men were observed in the high‐TMB group—a result consistent with recent reports suggesting potential associations of higher TMB in male patients.^46^ Additionally, baseline C‐reactive protein (a factor known to be associated with immune‐related AEs) was 24% higher at the median in patients with high‐tTMB than in patients with low‐tTMB.^47^ No clear imbalance in other prognostic factors were observed apart from TC2/3 expression and CRP. For this reason, logistic regression for efficacy and safety endpoints were performed using tTMB as the only predictor.

The majority of patients (≈94%) were treated with a 1200‐mg q3w dose of atezolizumab selected based on prior nonclinical and clinical data from several trials and approved indications.^32^, ^38^ This regimen was selected to provide adequate exposure to safeguard against the potential impact of anti‐drug antibody response and inter‐individual variability. We capitalized on a previously developed popPK model and did not re‐estimate PK model parameters in this study; however, given our external validation was acceptable, re‐estimation was not deemed necessary, and the Bayesian post hoc estimation of individual parameters was also considered acceptable. Exposures of atezolizumab after single and multiple doses were in line with those observed in prior assessments.^32^ Patients with high tTMB achieved an ORR approximately two‐fold higher than patients with low tTMB—a finding that did not appear to be driven by a single tumor type. A flat exposure‐response relationship was identified in the tTMB‐high population, both in the number of clinical responders (patients with CR + PR) and across the change in SLD over time. A comparable safety profile was seen in patients with high tTMB and in the entire tTMB‐evaluable population with grade 3/4 AEs; there were some numerical differences indicating a slightly higher incidence of all‐grade AESIs in the high‐tTMB population, but these rates were comparable to those in patients treated with other anti–PD‐L1/PD‐1 agents.^48^ We note that these observations might be related to improved efficacy in the high‐tTMB population—slightly higher AESI incidences could normally be expected with longer treatment duration. No relationship between AEs and atezolizumab exposure was seen in the tTMB‐high population, consistent with our knowledge of a flat exposure‐safety profile of atezolizumab.^32^, ^49^ Our exploratory tTMB‐response results agree both with prior analyses that revealed there were higher response rates in patients with higher tTMB tumors and with reports of associations between immune‐related AE reporting and tTMB score,^11^, ^50^ although other meta‐analysis data reporting associations between TMB and efficacy did not find any associations between TMB and toxicity.^51^ These observations will be evaluated prospectively in the MyPathway trial (ClinicalTrials.gov ID, NCT02091141). In patients treated with PD‐L1 or PD‐1 inhibitors, differences in immune‐mediated AE frequencies could potentially be driven by T cells reacting to tumor antigens that are cross‐reactive against wild‐type protein in normal tissue.^52^ Lastly, the exploratory tTMB‐response assessments are independent of exposure at therapeutic doses based on our flat exposure‐response findings.

Our analysis had several limitations in determining the potential for using an anti–PD‐1/PD‐L1 therapy across a tTMB‐high pan–tumor type indication, these limitations may also be relevant to the broader field. Our analysis was completed retrospectively using existing clinical data. We limited our assessments in the study to the use of objective response, longitudinal SLD change, and DOR to evaluate the potential for TMB as a prognostic utility for ICI therapy. A more comprehensive evaluation that included progression‐free survival and overall survival could provide additional insights into the utility of TMB to guide ICI therapy in future assessments. Currently, a standardized TMB cutoff that defines high mutational burden in any specific tumor type or across multiple tumor types does not exist. Moreover, differing cutoffs and algorithms are used with the multitude of TMB assessment platforms currently on the market. Determining a cutoff that can capture high mutational burden across a diverse set of tumor types is complex and highly dependent on tumor biology and the platform being used.^53^, ^54^ Also, it is possible that TMB values vary between tumor tissues, which could affect ICI treatment stratification. Recent findings report a bias for significantly higher TMB in metastatic tissue than in the primary tumor, although effectiveness in treatment benefit following ICI between both sources was considered comparable.^55^ Overcoming these obstacles is vital, and several national and regional US initiatives to harmonize TMB for reliable and reproducible use as a clinical biomarker of response to ICIs are underway.^56^, ^57^ Paving the way toward broader use of tTMB will necessitate leveraging data across a combination of clinical trials, flexibility in approaches, and multidisciplinary efforts to further advance tTMB as a diagnostic, therapeutic, and predictive biomarker of ICI benefit.^58^ The MyPathway trial will evaluate these prospectively and is adequately powered to address some of the limitations herein.

In summary, this article enhances our knowledge of complex predictive biological factors affecting response to atezolizumab. The pooled analysis revealed a positive benefit‐risk profile with higher response rates and longer duration of response achieved in patients with tTMB‐high tumors that supports the use of a 1200‐mg, every‐3‐week regimen of atezolizumab in a tumor‐agnostic high‐tTMB population. Safety was consistent with the known safety profile of atezolizumab. Exposures of atezolizumab were in line with expectations, with no exposure‐safety or exposure‐efficacy relationships identified. Prospective investigations are warranted to expand the inquiry to larger populations across diverse tumor types.

## DISCLOSURES

All authors disclose medical writing support funded by F. Hoffmann‐La Roche Ltd. All authors are employees of Genentech, Inc (part of the Roche Group), F. Hoffmann‐La Roche Ltd., or Roche Products Ltd. and are stockholders of F. Hoffmann‐La Roche Ltd.

## AUTHORS’ CONTRIBUTIONS

CSS, FAL, and DSS contributed to conception and design. CSS, PC, FAL, DSS, and RB contributed to development of methodology. SV, XH, and WZ contributed to acquisition of data. CSS, PC, FAL, DSS, RB, MDT, JS, and LB contributed to analysis and interpretation of data. All authors contributed to writing, review and/or revision of the manuscript. All authors approved the final manuscript and agreed to be accountable for all aspects of the work. Administrative, technical, or material support: NA. Study supervision: NA. Other: NA.

## PRIMARY LABORATORY OF ORIGIN

The analyses conducted in this paper are based on pooled data from several studies that were overseen by a number of principal investigators not included as authors given the exploratory nature of these analyses.

## Supporting information

Table S1–S3‐Figure S1–S7

## Data Availability

Qualified researchers may request access to individual patient level data through the clinical study data request platform (https://vivli.org/). Further details on Roche's criteria for eligible studies are available here (https://vivli.org/members/ourmembers/). For further details on Roche's Global Policy on the Sharing of Clinical Information and how to request access to related clinical study documents, see here (https://www.roche.com/research_and_development/who_we_are_how_we_work/clinical_trials/our_commitment_to_data_sharing.htm).
