# Supplementary material for: Pan‐cancer population pharmacokinetics and exposure‐safety and ‐efficacy analyses of atezolizumab in patients with high tumor mutational burden
Source: Pharmacol Res Perspect. 2020 Nov 25;8(6):e00685. doi: 10.1002/prp2.685 (PMC7689240; doi:10.1002/prp2.685)
Supplement: Supplementary file 1 — Table S1–S3‐Figure S1–S7 [file PRP2-8-e00685-s001.docx]

**Supplementary Data**

**Supplementary Table S1.** Studies and patients contributing to the analysis.

| **Study** | **Tumour**  **type** | **Phase** | **Design  (treatment setting)** | **No. of patients** | | | | |
| --- | --- | --- | --- | --- | --- | --- | --- | --- |
|  |  |  |  | **Treated, n** | **BEP, n** | **tTMB <16 mut/Mb, n** | **tTMB ≥16 mut/Mb, n** |  |
| Total | Pooled |  |  | 2894 | 986 | 811 | 175 |  |
| GO28753 (POPLAR) | NSCLC | II | Randomised  (2L/3L) | 142 | 14 | 9 | 5 |  |
| GO28915 (OAK) | NSCLC | III | Randomised  (2L+) | 422 | 180 | 140 | 40 |  |
| GO28754 (BIRCH) | NSCLC | II | Single arm  (1L+/2L+) | 659 | 101 | 70 | 31 |  |
| GO28625 (FIR) | NSCLC | II | Single arm  (1L+/2L+) | 137 | 47 | 40 | 7 |  |
| GO29293 (IMvigor210) | mUC | II | Single arm  (1L/2L+) | 429 | 141 | 115 | 26 |  |
| GO29294 (IMvigor211) | mUC | III | Randomised  (2L+) | 459 | 259 | 215 | 44 |  |
| PCD4989g^a^ | Pan-tumour | Ia/Ib | Multicohort  (advanced) | 646 | 244 | 222 | 22 |  |

1L, first line; 2L, second line; 3L, third line; BEP, biomarker-evaluable population; mUC, metastatic urothelial carcinoma; mut/Mb, mutations per megabase; NSCLC, non-small cell lung cancer; tTMB, tissue tumour mutational burden.

^a^Patients included were administered 1200 mg, 10 mg/kg, 15 mg/kg, or 20 mg/kg of atezolizumab every 3 weeks as monotherapy.

**Supplementary Table S2.** Tumour types from tTMB-evaluable patients.

| **Tumour type** | **No. of patients** | |
| --- | --- | --- |
|  | **tTMB <16 mut/Mb (≥16 tumour types)** | **tTMB ≥16 mut/Mb (≥8 tumour types)** |
|  |  |  |
| UC (bladder) | 330 | 70 |
| Breast (non-TNBC) | 9 | – |
| Breast (TNBC) | 82 | – |
| CRC | 10 | – |
| Endometrial | 10 | 1 |
| Oesophageal | 1 | – |
| Gastric | 11 | – |
| Head and neck | 16 | 1 |
| Haematologic | 1 | – |
| Melanoma | 11 | 12 |
| NSCLC | 259 | 83 |
| Other | 33 | 5 |
| Ovarian | 6 | – |
| Pancreatic | 1 | – |
| RCC | 30 | 2 |
| Soft tissue sarcoma | 1 | – |
| Squamous cell of the skin | – | 1 |

CRC, colorectal cancer; mut/Mb, mutations per megabase; NSCLC, non-small cell lung cancer; RCC, renal cell carcinoma; TNBC, triple-negative breast cancer; tTMB, tissue tumour mutational burden; UC, urothelial carcinoma.

**Supplementary Table S3.** Tumour types of responding patients by tTMB subgroup

| **Tumour type with indicated tTMB status** | **No. of responding  patients, n (%)** | **No. of complete responders, n (%)** |
| --- | --- | --- |
| **tTMB ≥16 mut/Mb** | | |
| UC | 23 (33) | 7 (10) |
| Endometrial | 1 (100) | 0 |
| Melanoma | 5 (42) | 2 (17) |
| NSCLC | 23 (28) | 3 (4) |
| **tTMB <16 mut/Mb** | | |
| UC | 52 (16) | 17 (5) |
| CRC | 1 (10) | 0 |
| Head and neck | 2 (13) | 0 |
| NSCLC | 41 (16) | 5 (2) |
| Other | 1 (3) | 0 |
| Ovarian | 1 (17) | 0 |
| RCC | 3 (10) | 1 (3) |
| TNBC | 8 (10) | 3 (4) |

Tumour types for non-responding patients are not shown. CRC, colorectal cancer; NSCLC, non-small cell lung cancer; mut/Mb, mutations per megabase; RCC, renal cell carcinoma; TNBC, triple-negative breast cancer; tTMB, tissue tumour mutational burden; UC, urothelial carcinoma (bladder).

**
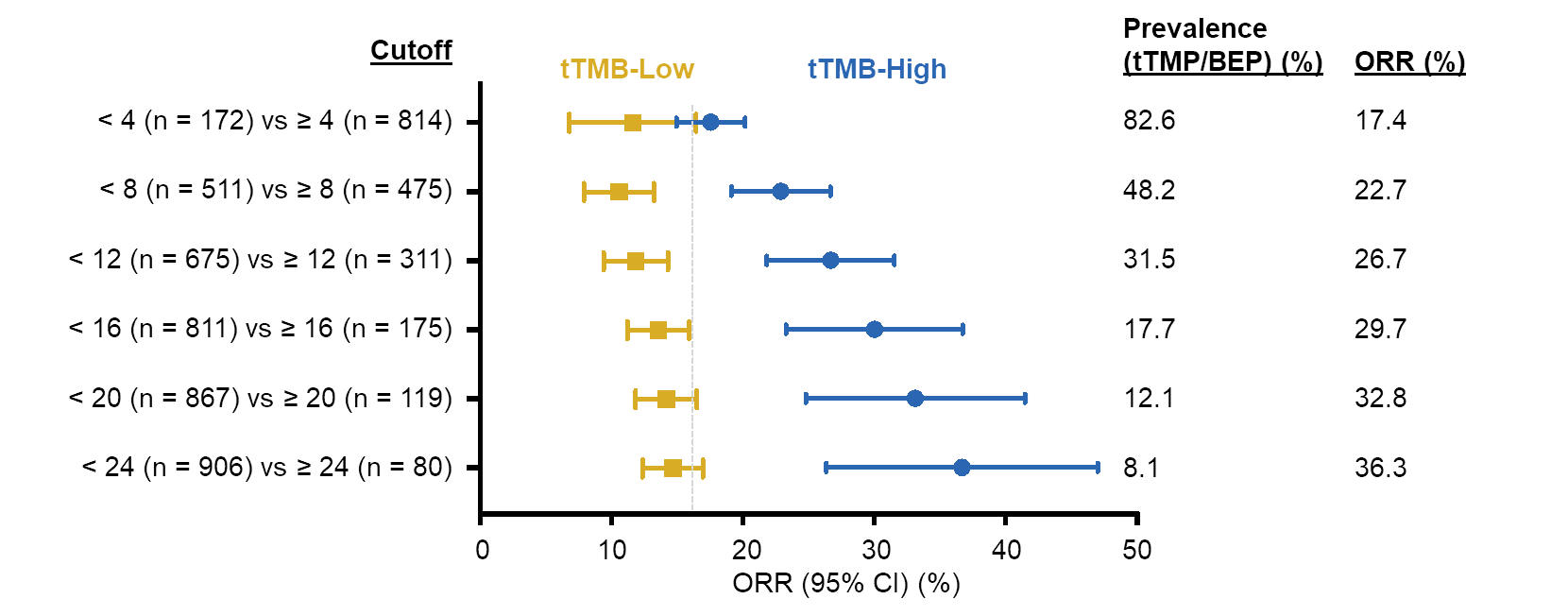
**

**Supplementary Figure S1.**

Objective response by tTMB. Forest plot of RECIST 1.1 ORR based on retrospective evaluation of tTMB at indicated cutoffs in pooled population of biomarker-evaluable patients treated with atezolizumab. tTMB levels are in mutations/Mb. ORR, objective response rate; RECIST, Response Evaluation Criteria in Solid Tumors; tTMB, tissue tumour mutational burden.


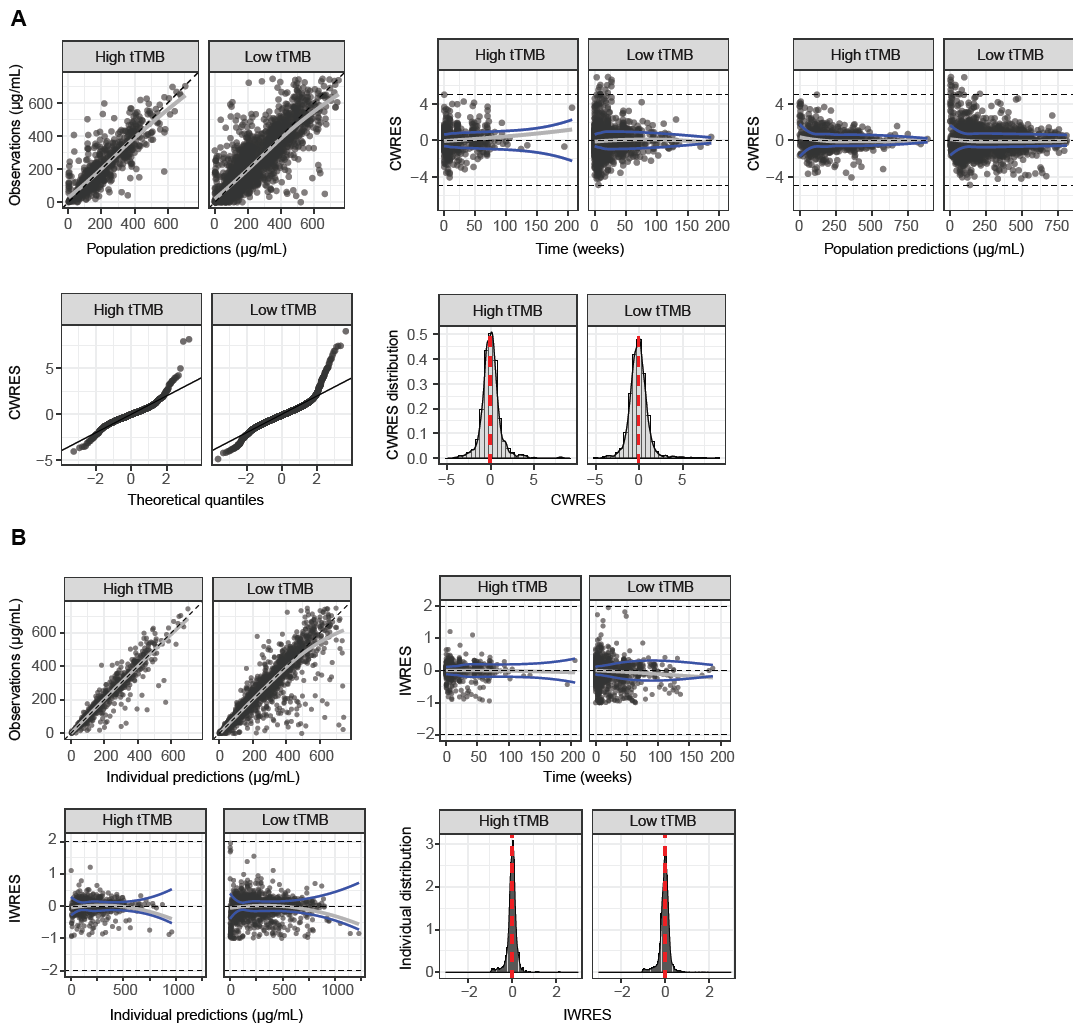
**Supplementary Figure S2.**

Population pharmacokinetic model goodness-of-fit diagnostic plots in tTMB-evaluable patients, stratified by low (tTMB <16 mut/Mb) or high (tTMB ≥16 mut/Mb) tTMB ([**A]** CWRES; [**B]** IWRES). Gray lines represent LOESS, blue lines represent LOESS in positive or negative residuals, and diagonal dashed lines represent lines of identity. CWRES, conditional weighted residuals; IWRES, individual weighted residuals; LOESS, locally weighted scatterplot smoothing; mut/Mb, mutations per megabase; tTMB, tissue tumour mutational burden.


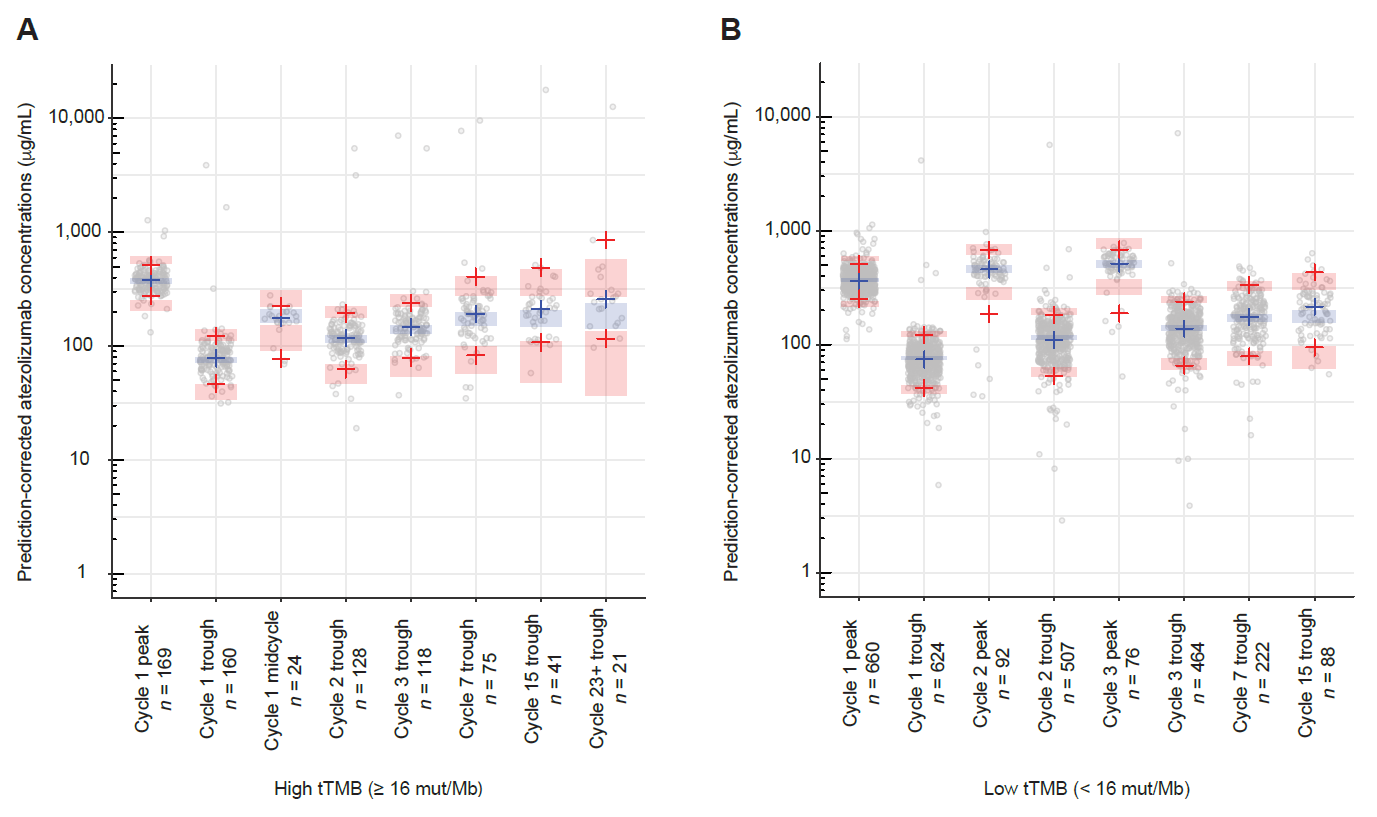


**Supplementary Figure S3.**

Population pharmacokinetic model prediction-corrected visual predictive check in TMB-evaluable patients, stratified by **(A)** high (tTMB ≥16 mut/Mb) or **(B)** low (tTMB <16 mut/Mb) TMB. Blue crosses and shaded areas represent the 50th percentiles of observed data and 95% prediction intervals of simulated data, respectively; red crosses and shaded areas represent the 5th and 95th percentiles of observed data and 95% prediction intervals of simulated data, respectively. Gray dots represent observed data, and n is the number of samples. Nine observations <1 µg/mL in the low-tTMB group are not shown. mut/Mb, mutations per megabase; TMB, tumour mutational burden; tTMB, tissue tumour mutational burden.


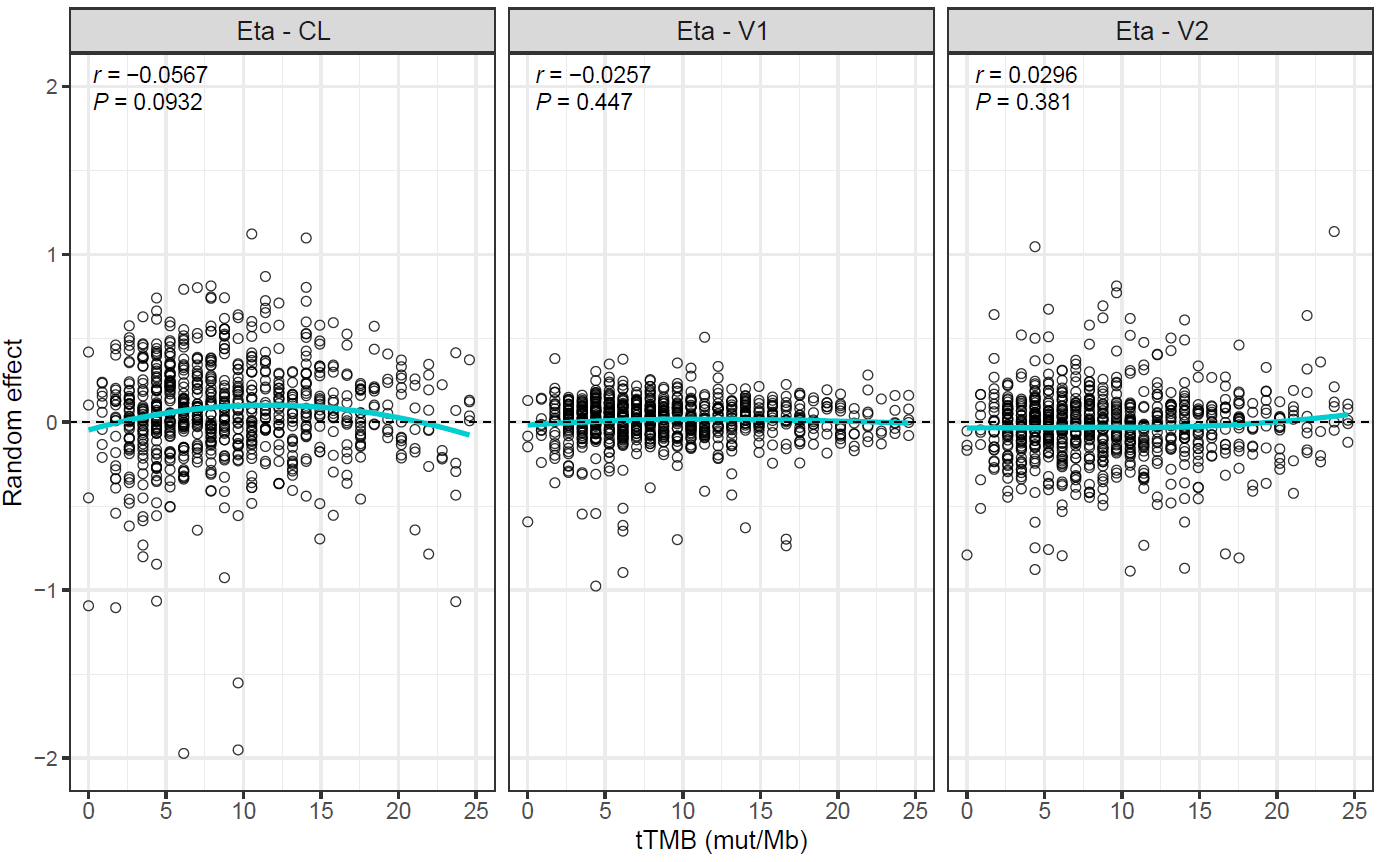


**Supplementary Figure S4.**

Inter-individual variability relationship between random effects of central clearance, central volume of distribution, and peripheral volume of distribution in the final population pharmacokinetic model using tTMB as a continuous variable. Analyses were adjusted for covariates in the model: albumin, body weight, tumour burden, and anti-drug antibodies. CL, central clearance; Eta, difference between value of parameter in individual and population; V1, volume of distribution of the central compartment; V2, volume of distribution of the peripheral compartment.


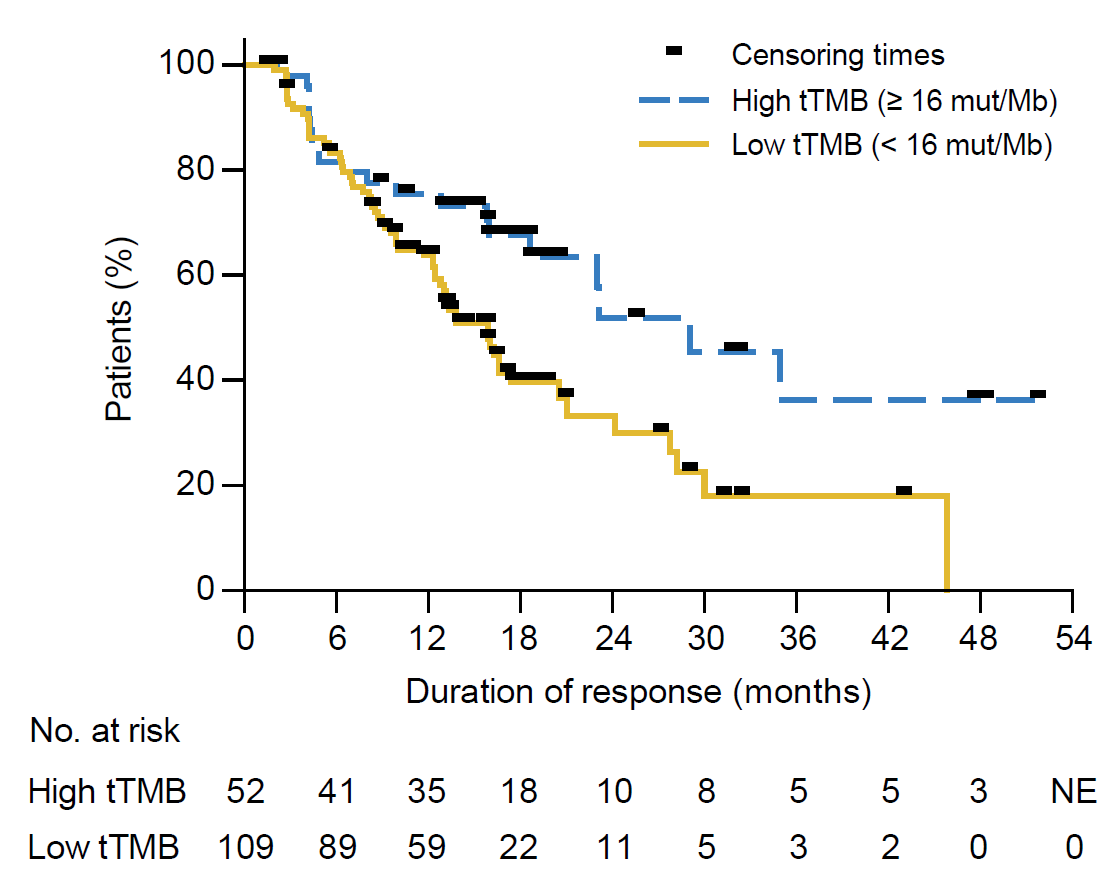


**Supplementary Figure S5.**

Kaplan-Meier plot for duration of response across 161 tTMB-evaluable responding patients. In the low-tTMB subgroup (n = 109), 63 events occurred; In the high-tTMB subgroup (n = 52), 20 events occurred. mut/Mb, mutations per megabase; tTMB, tissue tumour mutational burden.


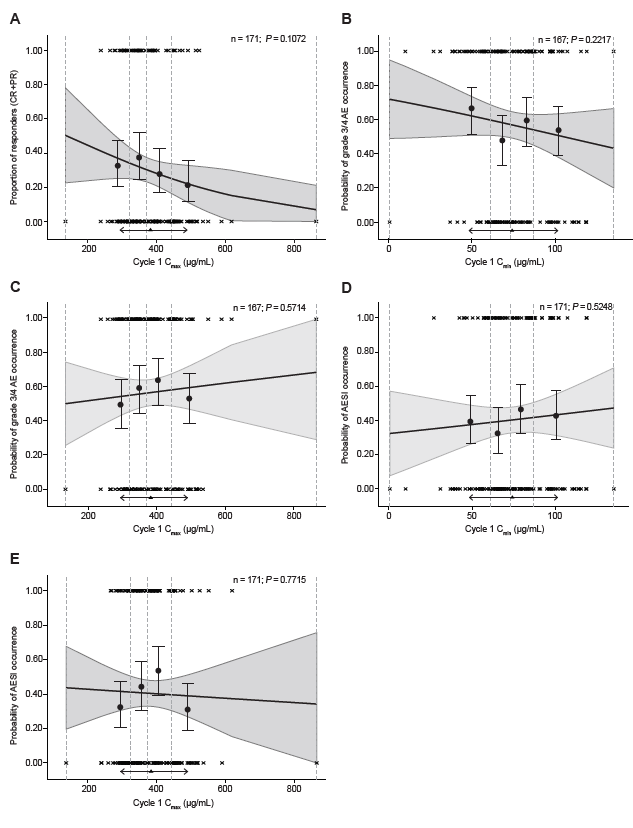


**Supplementary Figure S6.**

Proportion of tTMB-high patients who were responders (CR + PR) to atezolizumab by **(A)** cycle 1 C_max_, the proportion of tTMB-high patients with grade 3/4 AEs by **(B)** cycle 1 C_min_ (**C**) and C_max,_ and any-grade adverse event of special interest by (**D**) cycle 1 C_min_ and **(E)** C_max_. C_max_ and C_min_ values for each response event (yes, 1.00; no, 0) are represented by x symbols. Solid circles with standard error bars: proportion of response from binned observations by quartiles of the log-transformed exposure (*y* value); median exposure value within the bin (*x* value). Black line: model-fitted curve of the probability of response across atezolizumab exposure. Dashed lines: binning boundaries. Shaded area: 95% confidence band for the logistic regression curve. AE, adverse event; C_max_, maximum concentration; C_min_, minimum concentration; CR, complete response; PR, partial response; ORR, objective response rate; tTMB, tissue tumour mutational burden.


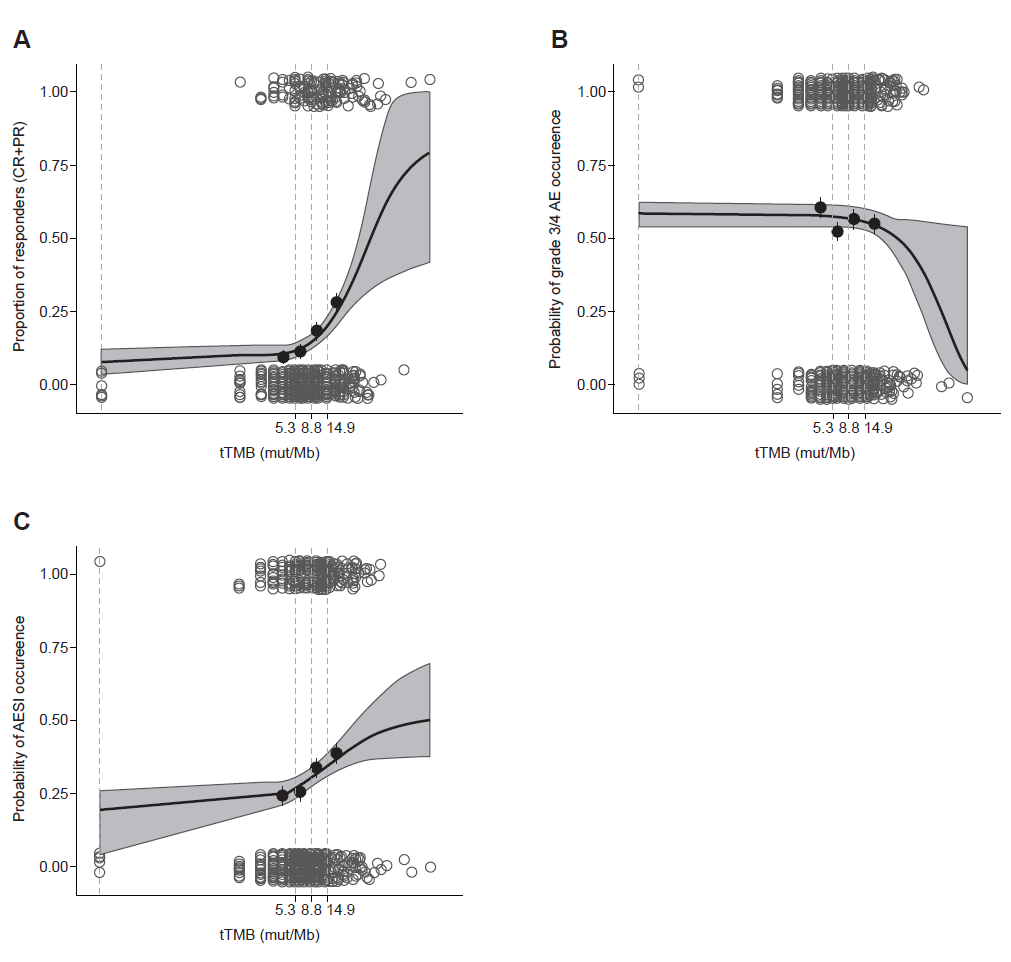


**Supplementary Figure S7.**

Exploratory TMB-response assessment. Proportion of tTMB-evaluable patients who **(A)** were responders (CR+PR) to atezolizumab by tTMB (mut/Mb) (**B**) had a grade 3/4 AEs and (C) had an any-grade AESI by tTMB (mut/Mb). Values for each response event (yes, 1.00; no, 0) are represented by open grey circles. Solid black circles with standard error bars: binned proportion of response from observations (*y* value); median TMB within the bin (*x* value). Black line: mean model-fitted curve for each TMB record in the data set). Dashed lines: binning boundaries. tTMB levels are binned based on the quartiles of the log-transformed tTMB levels. Shaded area: 95% confidence band for the logistic regression curve. Observed data points are based on n = 986, n = 951, and n = 986 patients in panels A, B, and C, respectively. AE, adverse event; AESI, AE of special interest; mut/Mb, mutations per megabase; TMB, tumour mutation burden; tTMB, tissue TMB.
